# Assessment of Knowledge of Health Economics among Healthcare Professionals in the Kingdom of Saudi Arabia: A Cross-Sectional Study

**DOI:** 10.3390/healthcare12020185

**Published:** 2024-01-12

**Authors:** Esraa Dhaif Allah Algharibi, Bodour Ayman Fadel, Mohammed Khaled Al-Hanawi

**Affiliations:** 1 Department of Health Services and Hospitals Administration, Faculty of Economics and Administration, King Abdulaziz University, Jeddah 21589, Saudi Arabia; 2 Health Economics Research Group, King Abdulaziz University, Jeddah 21589, Saudi Arabia

**Keywords:** health economics, health professionals, knowledge, professional practice, Saudi Arabia

## Abstract

Addressing the ongoing challenge of rising healthcare spending is crucial for ensuring the health quality of a population. At the core of healthcare systems, health professionals play a vital role in patient care and resource utilization. Despite healthcare cost concerns, health professionals often lack an understanding of health economics for optimal decision making. Accordingly, the aim of this study was to assess the knowledge of health economics among healthcare professionals in the Kingdom of Saudi Arabia. The broader goal was to identify knowledge gaps crucial for developing targeted interventions to maintain quality healthcare within the context of resource constraints. We used cross-sectional data collected from January to June 2023 and employed univariate, bivariate, and multivariable techniques for analysis. Univariate analyses were used to compare respondent proportions in socio-economic and demographic categories, bivariate analysis was used to examine the frequencies of independent variables related to the dependent variable, and a multivariate logistic regression model was used to identify the factors associated with knowledge of health economics among healthcare professionals. A total of 1056 responses were included for analysis. Approximately 35.35% of the sample possessed optimal knowledge of healthcare economics. Additionally, 58.14% of respondents considered health economics knowledge essential in their job practice, 16.95% regularly read articles on health economics, 22.06% engage in economic decision making at work, and 20.17% apply health economics techniques in their decision making. Health economics knowledge varied according to profession status, work experience, perceptions about health economics, and involvement in management tasks and decision-making processes. Generally, knowledge of health economics tended to increase with experience, positive perceptions, and engagement in administrative or management tasks. Nevertheless, knowledge of health economics is largely limited among health professionals in Saudi Arabia. Policymakers should address disparities in knowledge and perceptions of health economics through ongoing training courses and workshops. These interventions will ensure the presence of highly skilled professionals capable of implementing effective healthcare decisions and managing the increasing costs of healthcare.

## 1. Introduction

In recognizing healthcare as a fundamental human right, countries globally have enhanced the accessibility and coverage of healthcare services [1,2]. As healthcare significantly impacts individual economic well-being and productivity, strides have been made towards achieving high health quality for a population by improving the accessibility and affordability of healthcare services [3]. Improved health promotes economic growth by reducing productivity losses from illness and allowing the reallocation of resources from treatment to alternative endeavors [4]. The interplay among health, healthcare, and the economy has a bearing on the development trajectory of a country. As such, the global increase in health expenses has garnered attention from researchers seeking ways to optimize health and well-being amid limited resources [5].

Ensuring and maintaining health quality remain persistent challenges due to the ongoing rise in healthcare spending, involving increased costs across out-of-pocket, insurance, and hospital expenses [6]. Healthcare expenditures can exceed 10% of the gross domestic product (GDP) [7], with more extreme allocations in certain countries such as Iran, where 40% of the total health budget is allocated to hospital services [8]. The healthcare market in the Gulf region experienced an annual 11% increase in value from 2010 to 2014 [9]. In the Kingdom of Saudi Arabia (KSA), healthcare holds a significant share of the national budget. The increasing demand for healthcare is driven by factors such as an aging population, the rising prevalence of infectious and non-communicable diseases, advancements in technology and medical interventions, as well as the expansion of primary care and the growth of day surgery [10]. Rising healthcare costs pose a universal challenge, prompting global policymakers to seek economically sustainable healthcare solutions.

Health economics is a branch of economics that focuses on optimizing the allocation of resources in the healthcare sector to maximize population health [11]. Emerging after Arrow’s seminal work in 1963, health economics is based on classic economic principles, models, and techniques for healthcare decision making [12,13], specifically aiming to assess the quantity, quality, and value of limited resources to aid decisions that enhance health outcomes. The attributes considered to measure health economics include resource allocation, cost-benefit analysis, cost-effectiveness, and opportunity cost related to healthcare financing and interventions [14]. Despite significant government intervention, uncertainty, risk, asymmetric information, and externalities in the health sector, health economics can facilitate achieving efficiency in resource allocation for health purposes, while maximizing the impact of preventive and curative health services for the population [15].

Medical schools worldwide are increasingly incorporating health economics education, emphasizing economic evaluations to measure and compare the costs and benefits of healthcare interventions [16]. Additionally, there is a rising application of pharmaco-economics, which involves studying the impact of drug therapy costs on society and healthcare systems and comparing them with the value of economic healthcare outcomes [17]. Health economic evaluations enhance the healthcare decision-making process by supplying evidence to either challenge or support current allocations, enabling the reallocation of resources to interventions that yield the highest health benefits [18]. Health professionals constitute the core of healthcare systems, as the agents playing a crucial role in patient care and shaping the use of medical resources. Health professionals, particularly physicians and nurses, oversee significant budgets, constituting a substantial portion of healthcare expenditures [5].

Physicians and nurses with knowledge of health economics can enhance healthcare effectiveness through improved resource allocation and cost-effectiveness. In the high-cost healthcare setting of the United States, understanding health economics aids informed decision making, ensuring optimal clinical outcomes with consideration for financial implications [19]. In the United Kingdom, the National Institute for Health and Care Excellence (NICE) evaluates healthcare intervention cost-effectiveness [20,21]. Knowledge of health economics enables healthcare professionals to contribute to evidence-based NICE guidelines, thereby promoting effective resource use in the National Health Service (NHS) [22,23]. In Germany’s social health insurance system, health economics knowledge assists physicians and nurses in assessing the cost-effectiveness of treatments [24,25]. This enables collaboration with insurance companies to ensure patients receive suitable care within the insurance system [22,26].

Awareness of health economics varies significantly among physicians and nurses in both advanced and developing countries. In Israel, only 42% of physicians prioritize cost consciousness [27]. In the United Arab Emirates, only 3.6% of pharmacists show a strong understanding of pharmaco-economics [28]. In Jordan, nursing students exhibit higher health economics knowledge than dentistry students [29]. Meanwhile, 88% of Ethiopian medical professionals [30] and 92.8% of Indian doctors [31] believe that integrating health economics into curricula would enhance healthcare performance. In Tanzania, health professionals with managerial roles consider health economics to be more crucial than those solely in medical practice [32]. In general, healthcare professionals worldwide tend to have limited knowledge of health economics, particularly with respect to efficient resource allocation and optimization.

The challenge posed by conflicting demands between patient care and cost management is a contributing factor to the limited knowledge of health economics. Physicians grapple with the challenge of upholding high-quality patient care standards while simultaneously reducing the costs of services provided. For example, Storesund and McMurray [33] discovered that nurses tend to neglect the costs associated with clinical activities, and Logaraj et al. [31] reported a limited understanding among physicians regarding health economic concepts. However, with increasing healthcare expenditure, health professionals must identify not only the most effective treatments but also those that are cost efficient [16]. Thus, ensuring that medical professionals have a strong grasp of health economic concepts has the potential to enhance the overall efficiency of the healthcare system [34].

There is a particularly high need to optimize healthcare in the Kingdom of Saudi Arabia (KSA), where healthcare constitutes a significant share of the national budget; moreover, approximately 56% of the workforce relies on insurance and out-of-pocket health expenditures [10,35]. Additionally, the public healthcare services in the KSA face the challenges of being overburdened and overcrowded [36]. Applying health economics principles is essential for achieving efficiency in healthcare. However, accomplishing such efficiency is challenging without involving health professionals in decision-making and cost-management activities [37]. Therefore, the aims of this study were to assess the knowledge of health economics among healthcare professionals in the KSA. The participants included a diverse range of professionals such as physicians, dentists, pharmacists, nurses, healthcare management staff, and allied health professionals. We employed cross-sectional data, which were analysed through univariate, bivariate, and multivariable techniques to assess the knowledge of health economics among these healthcare professionals. This assessment is crucial for identifying gaps in knowledge and developing targeted interventions to ensure that health professionals are well-versed in health economics concepts, enabling quality care provision within the context of resource constraints.

## 2. Materials and Methods

### 2.1. Study Design and Sample

This cross-sectional study was conducted in Saudi Arabia, from 13 January 2023 to 26 June 2023, to assess the knowledge of health economics among healthcare professionals, including physicians, dentists, pharmacists, nurses, healthcare management staff, and allied health professionals. The data for this study were collected online via a self-administered questionnaire using SurveyMonkey (Ottawa, ON, Canada). The survey’s link was shared with healthcare professionals through WhatsApp groups to engage wider participation. In addition, participants were asked to forward the survey link to their colleagues. Based on the most recent statistics, there are 631,418 healthcare professionals working in the healthcare sector in the KSA [38]. The sample size required for this study was calculated using a sample size calculator [39], considering a margin of error of ±5%, a confidence level of 95%, a response distribution of 50%, and the estimated population size indicated above, resulting in a required sample size of 384 respondents.

### 2.2. Measurement Tool

The online self-administered questionnaire was designed and developed based on similar previous studies that aimed to assess the knowledge of health economics among different healthcare professionals [29,30,40,41,42]. The questionnaire was originally designed in English and translated into Arabic; the Arabic text was used to administer the survey. The questionnaire was piloted utilizing a sample of 30 participants (healthcare professionals) from the public and private healthcare sectors to ensure the face and content validity of the Arabic-translated questionnaire. In addition, the questionnaire was revised by two professors who specialized in health economics from King Abdulaziz University before the final version was used in the current study.

On the first page of the online questionnaire, respondents were provided with clear information about the context and objective of the study. Respondents were notified that they could choose to end the survey at any point, without needing to provide a reason, and that all information and opinions provided would be treated with full anonymity and confidentiality. To be eligible for participation, respondents had to be residents of Saudi Arabia, aged 18 years and above, understand the questionnaire’s content, and agree to take part in the study. Prior to proceeding with the questionnaire, participants were asked to provide online informed consent, indicating their agreement to participate in the study.

The questionnaire comprised three primary sections. The first section aimed to collect data on the socio-demographic characteristics and general information of the respondents. This included variables such as gender, employment status (governmental health sector employee or private health sector employee), specialty, type of activity they perform in their work, and years of experience. The second section focused on gathering information regarding participants’ awareness, practices, and experiences related to health economics. The questions in this section comprised items related to their involvement in economic decision making at work, their perspective on the importance of health economics knowledge in their job practice, their utilization of health economics techniques for decision-making, and their regular reading of articles pertaining to health economics. The third section assessed participants’ knowledge of health economics with 16 items and definitions covering various aspects of health economics, including overall health economics concepts and specific definitions such as opportunity cost, utility cost, direct cost, indirect cost, intangible cost, overhead cost, recurrent cost, discounting, quality services, GDP, marginal cost, technical efficiency, allocative efficiency, cost-effectiveness, benefit/cost analysis, and economic outcomes. For each item, the participants indicated whether the definition was true, false, or if they did not know. This was used to obtain a final score of their knowledge of each of these classic health economics concepts.

### 2.3. Dependent and Independent Variables

The dependent variable was the overall knowledge score on health economics based on the answers to the 16 true/false items in the survey: a score of zero was assigned to incorrect or uncertain answers (‘don’t know’), while correct responses received a score of one. The total knowledge score ranged from 0 to 16. A score of ≥ 50% (≥8 correct responses) was considered to reflect optimal knowledge.

Socio-demographic characteristics and general information of the respondents were used as independent variables. These variables included gender, employment status (governmental health sector employee or private health sector employee), specialty, type of activity they perform in their work, and years of experience. Gender was coded as 1 for men and 0 for women. The type of healthcare sector was divided into two categories: governmental health sector employee (reference category) and private health sector employee. The specialty variable was divided into six categories: allied health personnel (reference category), physician, dentist, pharmacist, nurse, and healthcare management staff. The type of activity performed at work was divided into three categories: mainly clinical (reference category), mainly managerial, and a mix of clinical and managerial. Total years of experience were divided into four categories: 1–5 years (reference category), 6–10 years, 11–15 years, and >15 years. Other independent variables, including whether participants incorporate economic decisions in their work, whether they believe that knowledge of health economies is necessary at their job practice, whether they read articles related to health economics regularly, and whether they apply health economics techniques at their work to make decisions, were all coded as 0 for ‘no’ and 1 for ‘yes’.

### 2.4. Statistical Analysis

The data analysis primarily involved univariate and multivariable regression techniques. Initially, univariate analyses were conducted to compare the proportions of respondents in each socio-economic and demographic category. Next, bivariate analysis was performed to compare the frequencies associated with different independent variables in relation to the dependent variable (i.e., knowledge score on aspects of health economics). This analysis involved cross-tabulation using the Chi-square test to determine any associations between the variables.

To assess the independent associations of each socio-economic factor with knowledge of health economics, a multivariate logistic regression model was utilized. Therefore, a multivariable logistic regression analysis was conducted to identify the factors related to knowledge of health economics among healthcare professionals. All analyses were conducted using STATA software (Version 16,StataCorp LP, College Station, TX, USA).

## 3. Results

A total of 1107 participants completed the questionnaire. After excluding 51 respondents who did not provide complete answers for all the variables of interest, the final sample comprised 1056 participants. The mean health economics knowledge score among healthcare professionals was 5.229 (SD = 5.534, range: 0–16). Approximately 35.13% of the sample demonstrated optimal knowledge of health economics (answering at least 50% of the questions correctly).

Table 1 presents the summary statistics of the study population. More than half of the participants were men and over three quarters were governmental health sector employees. With respect to professions, approximately one quarter of the sample was represented by physicians and approximately one fifth of the sample was represented by healthcare management staff. More than half of the participants had ≥6 years of working experience. Moreover, more than half of the sample indicated a belief that knowledge of health economics is necessary for their job practice, whereas less than one quarter of the sample regularly read articles related to health economics participate in economic decisions at work or apply health economics techniques to make decisions.

Table 2 and Table 3 show the results of the bivariate and logistic regression analysis of the association between knowledge of health economics and socio-economic and demographic characteristics, respectively. There were significant associations between having optimal knowledge of health economics (i.e., more than 50% correct answers in the survey) and employment status and professions, with healthcare management staff exhibiting approximately 2.3 times higher likelihood of having optimal knowledge than allied health professionals. Moreover, there was a significant association between the optimal knowledge of health economics and the type of activity that the healthcare professional performs at work. Compared with that of professionals performing only clinical activities, the proportion of individuals exhibiting optimal knowledge of health economics was approximately 3.1 times and 2.1 times higher among healthcare professionals who perform mainly managerial activities and a mix of clinical and managerial activities, respectively. Moreover, the optimal knowledge of health economics was significantly positively associated with years of experience; healthcare professionals with ≥15 years of work experience were 2.2 times more likely to have optimal knowledge of health economics compared with those having ≤5 years of work experience. Additionally, optimal knowledge of health economics was significantly associated with the perception of the importance of knowledge of health economics in their job practice, reading articles related to health economics regularly, incorporating economic decisions in their work, and applying health economics techniques to make decisions.

## 4. Discussion

This study assessed the knowledge of health economics among healthcare professionals in the KSA. Consistent with the literature, we found that a relatively small percentage (approximately 35.13%) of the sample possesses adequate knowledge of health economics. Specifically, Wilf-Miron et al. [27] found that only 42% of physicians reported a heightened level of cost consciousness in their daily practice. In another context, Al-Hemyari et al. [28] reported that merely 3.6% of pharmacists demonstrated a strong understanding of pharmaco-economics. This knowledge deficit can be attributed to several factors, including the absence of health economics education in health professional training and a lack of interest among health professionals in management issues, leading to an exclusive focus on patient care [40]. Moreover, decades of limited focus on the potential contribution of medical personnel to resource allocation in healthcare has hindered the acquisition of health economics knowledge among health professionals [11]. Over time, initiatives have emerged to promote such knowledge among health professionals, involving the implementation of policies and interventions to address the existing knowledge gap [43].

This study also revealed that those with more years of experience demonstrated better knowledge of health economics. Similar results were reported by Lee [42], indicating that greater knowledge of health economics was linked to years of experience and higher-ranked positions. This is likely due to employed individuals being exposed to ongoing training courses and workshops that may include health economics content. Moreover, health professionals with more years of experience are more likely to engage in such activities and learning opportunities compared to those with fewer years of experience.

In terms of the significance of health economics, this study revealed that health professionals who perceive incorporating health economics as an essential component of their job practice are three times more likely to possess optimal knowledge of health economics compared to those who do not hold such a belief. This is likely because a perceived importance motivates professionals to acquire related knowledge. Hammad et al. [29] found that nursing students exhibited greater knowledge of health economics compared to dentistry students, mainly because the nurses expressed a desire to undergo relevant training, whereas the dentists did not. In separate studies, 88% of medical professionals [30] and 92.8% of doctors [31] expressed the belief that incorporating health economics into academic curricula would enhance the performance of healthcare delivery. This underscores the need for governments to promote the dissemination of information emphasizing the importance of health economics. This approach would improve the attitudes of health professionals, thereby increasing their likelihood of acquiring the necessary knowledge.

Likewise, we found that the healthcare professionals who indicated that they regularly read articles related to health economics demonstrated twice the likelihood of having optimal knowledge compared to that of non-readers. The proportion of health professionals engaging in such reading varies in the related literature. In Mexico, Rodríguez-Ledesma et al. [44] reported that 17% of physicians regularly read materials on health economics, whereas in Chennai city, India, Logaraj et al. [31] found that most medical professionals were not regular readers of health economics articles and 39.7% did not read such literature at all. Al Husaini [45] emphasized that acquiring knowledge through reading is crucial for the learning process of medical professionals. Despite time constraints and source barriers, it is imperative for health professionals to cultivate the habit of regular reading, particularly focusing on articles related to health economics.

This study further revealed that those primarily involved in managerial activities were more likely to have optimal knowledge of health economics compared to professionals who are mainly involved in clinical activities. Given the contrasting demands health professionals face, while balancing healthcare cost concerns with ethical standards in patient care, it is likely that professionals not engaged in managerial activities may prioritize patient care over acquiring knowledge of health economics [46]. For instance, previous studies indicated that nurses, particularly those not involved in administrative responsibilities, tend to prioritize patient care while overlooking hospital costs [47,48]. Health professionals with managerial responsibilities are more likely to perceive health economics as crucial and are therefore more inclined to read articles on the subject, resulting in greater knowledge compared to that of health professionals solely engaged in medical practice [32]. This previous work also clarifies our finding that the professionals involved in economic decision making and those who apply health economics techniques in their work had 1.3 and 1.9 times higher optimal knowledge on health economics, respectively, compared to that of their counterparts.

Furthermore, the results also revealed that healthcare management staff have a significantly higher likelihood of being knowledgeable about health economics compared to allied health professionals. This disparity calls for the need to involve health professionals in decision making related to healthcare delivery. Ntlabezo et al. [46] found that nurses working with nurse managers who maintained budgetary control demonstrated greater economic awareness than those who did not. Exposure to decision-making positions and tasks is likely to drive health professionals to actively seek activities for enhancing their knowledge in health economics, resource allocation, and optimal expenditure in decision-making processes. Given the current bureaucratic structure prevalent in most public hospitals, hindering contributions to decision making and organizational policies, there is a need for reforms to facilitate the active participation of health professionals in decision making.

Some key aspects in health economics are noteworthy to place these findings into perspective. First, decision making in the health sector is often influenced by factors beyond health economics and economic evaluations [16]; social, political, and economic contexts predominantly shape decisions in these realms [49,50]. Second, a large portion of economic evaluations are funded by the pharmaceutical industry with its own motives [18]. Moreover, not all interventions undergo economic evaluations that are applicable to a specific context, potentially resulting in biased decisions by decision-makers [16,41]. Lastly, the application of health economics knowledge by health personnel in daily practice is not universal [22,23]. Decision making on services and medications often occurs at regulatory levels, rendering such knowledge superfluous for physicians or high-level health personnel who must adhere to institutional or health system guidelines [20,21].

Certain limitations of this study must be acknowledged. In particular, the reliance on self-reported questionnaires introduces the potential for data inaccuracies and social-response bias, as participants may provide responses deemed socially desirable rather than reflective of their true knowledge of health economics. Despite these limitations, this study offers valuable insights into the current state of health economics knowledge among health professionals in the KSA. This information provides a foundation for the government to formulate strategies aimed at enhancing health economics knowledge, thereby contributing to the improvement of optimal healthcare provision.

## 5. Conclusions

Using univariate, bivariate, and multivariable analyses, this study assessed the knowledge of health economics among healthcare professionals in the KSA. The study achieved its objective by showing the level of knowledge of health economics through its assessment. The assessment indicates that while more than half of the respondents consider health economics knowledge essential in their job practice, only a small percentage possess optimal knowledge, engage in economic decision making at work, and apply health economic techniques in their decision making. Furthermore, health economics knowledge varies depending on professional status, work experience, perceptions about health economics, and involvement in management tasks and decision-making processes. Generally, knowledge of health economics tends to increase with experience, positive perceptions, and engagement in administrative or management tasks. These findings highlight the need for policymakers to address existing limitations and disparities in knowledge and perceptions of health economics through ongoing training courses and workshops. The full benefits of health economics may be realized when the healthcare workforce is fully informed and engaged in economic decision making. Therefore, it is crucial to incorporate health economics training into the medical school curriculum before professionals enter the field. Additionally, the government must create platforms that allow health professionals to apply their knowledge of health economics in making cost-effective decisions regarding healthcare provision.

## Figures and Tables

**Table 1:** Summary statistics of the study population (N = 1056).

Variable	N	%
Gender		
Women	492	46.59
Men	564	53.41
Employment status		
Governmental health sector employee	834	78.98
Private health sector employee	222	21.02
Professions		
Allied health professional	248	23.48
Physician	261	24.72
Dentist	76	7.20
Pharmacist	83	7.86
Nurse	166	15.72
Healthcare management staff	222	21.02
Type of activity performed at work		
Mainly clinical	592	56.06
Mainly managerial	278	26.33
Mix of clinical and managerial	186	17.61
Experience		
1–5 years	485	45.93
6–10 years	272	25.76
11–15 years	129	12.22
>15 years	170	16.10
Consider knowledge of health economics to be important in job practice		
No	442	41.86
Yes	614	58.14
Read articles related to health economics regularly		
No	877	83.05
Yes	179	16.95
Participate in economic decisions at work		
No	823	77.94
Yes	233	22.06
Implement health economics techniques to make decisions		
No	843	79.83
Yes	213	20.17

**Table 2:** Bivariate analysis of optimal knowledge towards health economics and socio-economic and demographic characteristics.

Variable	Number	Proportion Exhibiting Optimal Knowledge (%) ^a^	Chi-Square
Gender			1.620
Women	492	33.13	
Men	564	36.88	
Employment status			8.103 ***
Governmental sector employee	834	37.29	
Private sector employee	222	27.03	
Professions			151.877 ***
Allied health professional	248	19.35	
Physician	261	30.27	
Dentist	76	27.63	
Pharmacist	83	27.71	
Nursing	166	27.71	
Healthcare management staff	222	69.37	
Type of activity performed at work			223.968 ***
Mainly clinical	592	15.88	
Mainly managerial	278	63.67	
Mix of clinical–managerial	186	53.76	
Experience			109.889 ***
1–5 years	485	20.41	
6–10 years	272	37.87	
11–15 years	129	51.16	
>15 years	170	60.59	
Consider knowledge of health economics to be important in job practice			158.293 ***
No	442	13.35	
Yes	614	50.81	
Read articles related to health economics regularly			110.239 ***
No	877	28.16	
Yes	179	69.27	
Participate in economic decisions at work			115.516 ***
No	823	26.73	
Yes	233	64.81	
Implement health economics techniques to make decisions			130.702 ***
No	843	26.69	
Yes	213	68.54	

^a^ Optimal knowledge was classified according to answering at least 50% of the knowledge questions correctly. *** *p* < 0.01.

**Table 3:** Association between optimal knowledge of health economics and socio-economic and demographic factors based on logistic regression analysis.

Variables	OR	95% CI
Gender		
Women	Reference	
Men	1.159	0.834–1.610
Employment status		
Governmental sector employee	Reference	
Private sector employee	0.889	0.575–1.376
Professions		
Allied health professional	Reference	
Physician	1.262	0.769–2.073
Dentist	1.624	0.814–3.238
Pharmacist	1.064	0.550–2.057
Nursing	0.996	0.570–1.740
Healthcare management staff	2.346 ***	1.327–4.145
Type of activity performed at work		
Mainly clinical	Reference	
Mainly managerial	3.100 ***	1.863–5.160
Mix of clinical and managerial	2.165 ***	1.383–3.390
Experience		
1–5 years	Reference	
6–10 years	2.022 ***	1.347–3.036
11–15 years	1.908 ***	1.154–3.152
>15 years	2.164 ***	1.341–3.492
Consider knowledge of health economics to be important in job practice		
No	Reference	
Yes	2.909 ***	1.989–4.252
Read articles related to health economics regularly		
No	Reference	
Yes	2.125 ***	1.362–3.318
Participate in economic decisions at work		
No	Reference	
Yes	1.315 ***	1.011–1.997
Implement health economics techniques to make decisions		
No	Reference	
Yes	1.942 ***	1.295–2.913
Constant	0.051 ***	
Observations	1056	
Pseudo R-squared	0.2813	
Chi-square	385.20 ***	

Abbreviations: CI, confidence interval; OR, odds ratio. *** *p* < 0.01.

## Data Availability

The datasets generated and/or analyzed during the current study are not publicly available due to privacy and confidentiality agreements as well as other restrictions but are available from the corresponding author (M.K.A.-H.) upon reasonable request.
